# Computational predictors fail to identify amino acid substitution effects at rheostat positions

**DOI:** 10.1038/srep41329

**Published:** 2017-01-30

**Authors:** M. Miller, Y. Bromberg, L. Swint-Kruse

**Affiliations:** 1 Department of Biochemistry and Microbiology, Rutgers University, 76 Lipman Dr, New Brunswick, NJ 08901, USA; 2 Department for Bioinformatics and Computational Biology, Technische Universität München, Boltzmannstr. 3, 85748 Garching/Munich, Germany; 3 TUM Graduate School, Center of Doctoral Studies in Informatics and its Applications (CeDoSIA), Technische Universität München, 85748 Garching/Munich, Germany; 4 Department of Genetics, Rutgers University, Human Genetics Institute, Life Sciences Building, 145 Bevier Road, Piscataway, NJ 08854, USA; 5 Institute for Advanced Study at Technische Universität München (TUM-IAS), Lichtenbergstraße 2a, 85748, Garching/Munich, Germany; 6 The Department of Biochemistry and Molecular Biology, The University of Kansas Medical Center, 3901 Rainbow Blvd, Kansas City, KS 66160, USA

## Abstract

Many computational approaches exist for predicting the effects of amino acid substitutions. Here, we considered whether the protein sequence position class – rheostat or toggle – affects these predictions. The classes are defined as follows: experimentally evaluated effects of amino acid substitutions at toggle positions are binary, while rheostat positions show progressive changes. For substitutions in the LacI protein, all evaluated methods failed two key expectations: toggle neutrals were incorrectly predicted as more non-neutral than rheostat non-neutrals, while toggle and rheostat neutrals were incorrectly predicted to be different. However, toggle non-neutrals were distinct from rheostat neutrals. Since many toggle positions are conserved, and most rheostats are not, predictors appear to annotate position conservation better than mutational effect. This finding can explain the well-known observation that predictors assign disproportionate weight to conservation, as well as the field’s inability to improve predictor performance. Thus, building reliable predictors requires distinguishing between rheostat and toggle positions.

Recent years have seen an explosion in the number of genomes and exomes sequenced for research and medical purposes, *e.g.* for diagnosing and prognosing predisposition to or progression of disease^1^,^2^,^3^. Unfortunately, our ability to interpret sequence data lags far behind. Take exomes, for example: Any given individual can have over 10,000 amino-acid differences in their protein coding regions, as compared to the reference genome^4^. These differences are often caused by nsSNPs (non-synonymous single nucleotide polymorphisms); in this manuscript, we refer to amino acid substitutions as *variants*. Given the large number of variants, it is not feasible to experimentally determine the *outcomes/effects* for all changes, *i.e.* how they alter molecular function, protein structure, evolutionary fitness, or lead to pathogenesis. Thus, dozens of computational algorithms have been developed to predict outcomes. However, current algorithms have significant room for improvement^5^.

Interestingly, underneath the assortment of computational/mathematical techniques and numerous data inputs, all variant-effect predictors rely, to various extents, on two broad concepts: (i) basic biological principles and (ii) pattern recognition techniques, optimized using results from wet-lab experiments. We examined the assumptions underlying these concepts, hoping to identify avenues to improve predictions.

The basic biological principles include biochemical amino acid similarities and evolutionary information about the protein of interest. The latter is usually in the form of a multiple sequence alignment (MSA) of homologs and is used to determine which specific sequence positions show evolutionary constraints (*i.e.* conserved *vs.* non-conserved). This information serves as a proxy to predict which amino acid substitutions are “allowed” (inferred by their presence in functionally-active homologs) and which are “bad” (selected against during evolution and, thus, absent from the multiple sequence alignment). Some variant-effect predictors use globally-applicable scoring matrices, *e.g.* BLOSUM^6^, that represent the likelihoods of amino acid substitutions in curated MSAs. The substitutions allowed by these matrices often match the biochemical classifications of side chains found in textbooks, which, in turn, are often used to design experimental mutation studies. By convention, only substitutions of “similar” amino acids are expected to allow normal or near-normal protein activity, while other substitutions are expected to alter or abolish function.

Variant-effect predictors that incorporate pattern recognition approaches are often training-driven. That is, they use large sets of experimentally verified functional effects of amino acid substitutions to build predictive models. These methods often (wrongly) assume that the variants used for training broadly represent the entire world of variation. Another extrapolation is implicit in any computational method that incorporates MSAs: if a particular variant-effect is known for one homolog, similar outcomes are expected for the same variant in other family members.

Both the conventional substitution rules and training datasets are biased by a gap between unbounded evolutionary reality and limited laboratory work; *i.e.* laboratory variants are subject to experimental limitations and to the interests of the scientists. Indeed, available experimentally-annotated sets of variants are heavily biased to the study of conserved amino acid positions^7^.

However, many protein positions are *not* conserved in MSAs; *i.e.* they *do* change during evolution. Non-conserved positions are often ignored as not important, but this need not be true: homologs often evolve functional variance *via* amino acid changes at non-conserved positions^8^ and disease-causing substitutions can occur at non-conserved positions^9^,^10^.

We hypothesized that key non-conserved positions follow different substitution rules than conserved positions. If so, prediction algorithms that rely on the conventional substitution rules or on laboratory-derived training sets might not correctly predict the variant outcomes at non-conserved positions. Indeed, we recently showed that some functionally-important, non-conserved positions do *not* follow *any* of the evolutionary or biochemical assumptions made for conserved positions^11^: In that work, we identified 12 positions in the LacI/GalR family of proteins that varied widely among family members^8^. Next, using the natural *E. coli* lactose repressor protein (LacI) and modified (synthetic) versions of seven LacI/GalR homologs (including GalR, PurR, and RbsR), we substituted the native amino acid in each of these positions with 5–13 other amino acids and measured functional outcomes^11^. If these positions were functionally important, the conventional rules would predict that only a few similar substitutions would allow normal function and that most others would abolish function (*e.g.* Fig. 1, left panel). However, at most of the chosen non-conserved positions, variants exhibited a wide range of functional effects (Fig. 1, right panel). Furthermore, these effects did not correlate with evolutionary frequency, side chain similarities, or functional effects of the same substitutions in homologous proteins^11^.

We named these positions *rheostats* after their most prominent characteristic: When multiple amino acids were substituted at one rheostat position, functions of the mutant proteins could be rank-ordered to show a progressive effect (Fig. 1, right panel). This contrasts with the *toggle* (on-off) behavior that is frequently observed at conserved positions and is predicted by the conventional rules (Fig. 1, left panel). We have also noted rheostatic behavior in published variant datasets for other proteins (*e.g.* refs 12, 13), which indicates that rheostat positions are likely widespread in the protein universe. Importantly, the progressive functional impact of variants at rheostat positions is all but disregarded by the current variant-effect predictors, which were either developed using variants at toggle positions and/or the thresholded (binary) version of functional outcomes. We also hypothesized that the poor correlation observed between functional outcomes of substitutions and biophysical/evolutionary amino acid properties could contribute to erroneous predictions.

Here we compare predictions from 16 widely-used computational methods for experimentally-evaluated substitutions at rheostat and toggle positions in LacI. Each variant at each position was assigned as either non-neutral or neutral based on experimental outcomes (Methods). When experimental outcomes were compared to predictions at rheostat positions, many non-neutral variants were incorrectly predicted to be neutral. At toggle positions, neutral variants were also poorly predicted, as previously observed by Gray *et al*.^7^. We gained insight into this problem by comparing the overall prediction ranges for rheostat and toggle positions, instead of using the default binary neutral/non-neutral thresholds: Our results suggest that current computational predictions could be enhanced by first determining whether the affected position functions as a rheostat or as a toggle.

Finally, our results show that evaluations of predictor performance are misled by bias in the available experimental data: As noted above, there is a dearth of experimental results for variants at rheostat positions; in addition, at toggle positions, the number of experimentally validated non-neutral variants is high and the number of validated neutral variants is very low. The latter is further complicated by an arguably obfuscated definition of “neutrality” that often differs among methods. Together, these biases have had the practical effect that all toggle variants appear to be non-neutral and all rheostat variants are assumed to be neutral. This leads to an artificially low number of variants incorrectly predicted as non-neutral, whereas the number of incorrect, neutral predictions cannot be properly estimated due to the low number of experimentally-validated neutral toggles. We propose that eliminating data bias, *e.g.* by using experimental results that annotate rheostat position variants, and optimizing prediction algorithms to account for position type will lead to more accurate evaluations of variant impact.

## Results

To test the performance of variant-effect predictors at rheostat and toggle positions, we extracted positions that exhibit these behaviors from two experimental datasets for LacI (Methods; Fig. 2)^11^,^14^. Rheostatic positions were identified by variants whose outcomes showed a progressive distribution; notably the outcomes of individual variants did not correlate with evolutionary frequency, side chain similarities, or outcomes in homologous proteins^11^. Rheostats were identified in both experimental datasets; for this work, predictions were compared to the quantitative measurements of Meinhardt *et al*.^11^. Toggle behavior was identified by *binary* variant outcomes, *i.e.* amino acid substitutions caused either a severe impact or no/weak effects; predictions were compared to data from the semi-quantitative study by the Miller lab^14^.

We designated three sets of rheostat and toggle variants (Methods, Supplemental Tables 1 and 2): (i) The *stringent* set comprised the nine rheostat positions that were identified by both experimental studies, and a comparable number of nearby toggle positions. (ii) The *complete* set comprised all 12 rheostat positions from the quantitative experiments and a comparable number of toggle positions. (iii) The *extended* set comprised all 12 rheostat positions and 50 toggles from the semi-quantitative study. For both rheostat and toggle positions, individual variants were classified as either neutral or non-neutral according to their experimentally-determined fold-change relative to wild-type repression.

For all variants in these sets, we predicted outcomes using 16 selected prediction algorithms (Fig. 3; Supplementary Tables 3 and 4). Most prediction trends were similar for all three sets (Fig. 4; Supplementary Figs 1, 2 and 3); differences are highlighted in the text below and likely stem from the small numbers of neutral variants at toggle positions (the *stringent* set contained only four neutral variants and the *extended* set contained 28). Experimentally-determined outcomes were compared to computational predictions in two ways: First, we compared the overall distributions for prediction scores from each of the four classes (rheostat non-neutral, rheostat neutral, toggle non-neutral, and toggle neutral). Second, for algorithms that generate continuous prediction scores, we directly compared predictions and experiments for individual variants.

### Differences between rheostat and toggle positions confound variant-effect predictions

Variant predictors report their results in one of two ways: as a binary decision (neutral/non-neutral) or as a scored value representing the likelihood of non-neutrality, often thresholded to make a binary decision. Ideally, each algorithm would clearly separate the distributions of neutral and non-neutral variants, regardless of their toggle or rheostat position location (Fig. 4a). That is, methods should differentiate toggle neutrals from both toggle and rheostat non-neutrals; moreover, rheostat neutrals should be classified similarly to toggle neutrals. Finally, the progressive effect of rheostat non-neutrals should be different from the binary effect of toggle non-neutrals (Fig. 4a).

However, for the *stringent* comparison set, no algorithm significantly distinguished toggle neutrals from toggle non-neutrals (Fig. 4b and Supplementary Fig. 1, (*B) vs. (D*): dark red vs. dark blue), as determined by the Kolmogorov-Smirnov test (KS-test) for continuous predictors and the Fisher exact T-test for binary predictors. The *extended* set was better differentiated at toggle positions by 8 of 16 methods (Supplementary Fig. 3). At rheostat positions in the *stringent* set, neutrals and non-neutrals were significantly differentiated by only three of the methods (SNAP2^15^, PROVEAN^16^, and PolyPhen-2^17^; Fig. 4b and Supplementary Fig. 1, (*A) vs. (C*), light red vs. light blue).

Note, however, that non-neutral variant PolyPhen-2 scores are nearly uniformly distributed over the entire score range. The *complete* and *extended* sets were differentiated by four methods (the three above and MutPred2, the unpublished successor of MutPred^18^; Supplementary Figs 2 and 3).

In contrast, toggle non-neutrals were well-differentiated from rheostat neutrals (Fig. 4b and Supplementary Fig. 1, (*B) vs. (C*), dark red vs. light blue) in all three comparison sets by all methods except PANTHER^19^. Note that PANTHER only returned predictions for 30% of all variants; no predictions were made for neutrals in the *stringent* or *complete* sets, and only 8 of 26 neutrals had predictions in the *extended* set. This makes PANTHER an outlier to most trends observed for all other methods. Some of the algorithms also correctly and significantly differentiated rheostat non-neutrals from toggle neutrals (Fig. 4b and Supplementary Fig. 1, (*A) vs. (D*), light red vs. dark blue). However, all of the continuous prediction methods erroneously assigned higher scores to toggle neutrals than to rheostat non-neutrals – the opposite of the correct prediction. Furthermore, for the larger *extended* set, the distinction between rheostat non-neutrals and toggle neutrals was eliminated (Supplementary Fig. 3); this finding, again, likely indicates the influence of the low number of toggle neutrals in the *stringent* set.

Finally, every method scored toggle neutrals (Fig. 4b and Supplementary Fig. 1, dark blue), on average, above the neutrality threshold and higher than rheostat neutrals (light blue), which on average scored below the neutrality threshold. This difference was significant for 12 of 16 methods. This trend was maintained for the larger comparison sets, suggesting that this issue is inherent to the prediction methods.

### Weak correlation between predictions and experimental outcomes at rheostat positions

The main goal of variant-effect prediction is to classify functional outcomes into binary neutral/non-neutral categories. Nevertheless, several predictors calculate continuous prediction scores, allowing us to correlate predictions with experimental effects for individual variants.

For rheostat positions, this would be a more valuable prediction, since the thresholds for biological effects can change with environment (*e.g*., in response to changes in other proteins or in cellular conditions)^20^,^21^. Thus, we examined whether the progressive nature of variant outcomes at rheostat positions was captured by the continuous score prediction methods for the *rheostat_9* and *rheostat_12* sets.

For the *rheostat_9* set, only 50% of the continuous prediction methods showed any correlation (Fig. 5; Supplementary Fig. 4). Moreover, only four of the sixteen methods (SNAP2, PROVEAN, MutPred2, and PolyPhen-2) showed statistically-significant differentiation of rheostat neutrals from non-neutrals in the *rheostat_9* or *rheostat_12* sets. Of these, SNAP2 exhibited the highest correlation (Pearson’s *r* = 0.58, *rheostat_9*; Fig. 5). Using the *rheostat_12* set did not alter the observed trends (Supplementary Table 5).

### Predictions for variants at *neutral positions* have unclear outcomes

The LacI/GalR experimental study carried out experiments at the same positions in multiple homologs^11^. When outcomes were compared, each rheostat position showed a rheostatic profile in ~80% of the homologs studied. Thus, rheostat behavior appears to be the baseline for these positions within the protein family, even though individual homologs may evolve the position to different roles. Positions 60 and 62 were among those that appear able to evolve new roles: These positions acted as rheostats in most of the homologs, but in LacI were neither toggle nor rheostat in either dimer or the tetramer datasets^11^,^14^. Instead, these were *neutral positions* – no variant had a significant effect on repression (in the absence of inducer). Note the distinction between a neutral position and a neutral substitution. Rheostat positions comprise both neutral and non-neutral substitutions, as long as the number of non-neutral variants greatly exceeds the number of neutral variants. Neutral positions comprise only neutral substitutions.

Here, most variants at neutral positions 60 and 62 were indeed predicted to be neutral (Fig. 5, set *rheostat* {*60, 62*}). However, the apparent success may be spurious for two reasons: First, our other current results show that all variants at rheostat positions generally score lower than those at toggle positions, and the results for positions 60/62 could just be a manifestation of that. Second, one of the correctly predicted neutral variants at position 62 was not inducible in experiments. In this work, we generally did not consider the variant effect on LacI induction, because for most variants, significant changes were not experimentally observed (Supplementary Table 2). However, altered response to inducer is classified as dramatic biological impact (the “+s” phenotype in the tetramer dataset)^14^. If this information were taken into consideration, the predictors would be considered to have failed for this non-inducible variant.

To resolve the performance of variant-effect predictors at neutral positions, we ideally need two additional experimental datasets: One comprising variants at positions 60/62 in other natural LacI/GalR homologs (the first study used synthetic homologs, as described in Methods below); and one comprising variants at other neutral positions in a variety of proteins.

## Discussion

We previously showed experimentally that amino acid substitutions at rheostat positions have different functional outcomes than those expected for toggle positions^11^. Here, we show that these differences impair the performance of variant-effect prediction algorithms. Overall, the predictors differentiated toggle positions from rheostat positions better than neutral variants from non-neutral variants. This may be due to the fact that, in LacI, rheostat positions are not conserved, whereas most toggle positions are. Many studies have noted that conservation is a key factor in identifying neutral and non-neutral variants^22^. Moreover, the rheostats host the majority of neutral variants. If these characteristics are common to other proteins, then our results demonstrate one possible reason for the disproportionate importance of conservation for existing tools and for the consistent lack of significant improvement of method performance.

Furthermore, we previously found that predictors disagreed in their annotation for roughly a fifth of the variants^15^,^23^. Here, we show that this number cannot be accounted for by mis-predictions at toggle positions: Most methods correctly predict toggle non-neutrals, and toggle neutrals are few in number, comprising a small percentage of the total predictions. Thus, mis-predicted rheostat variants are, arguably, a key factor that differentiates prediction methods.

Next, we considered the fact that threshold choice directly affects binary variant-effect predictions. Current variant-effect predictors use various input parameters to calculate a likelihood that a given, single amino acid substitution has an effect on protein function, disease relevance, structure, etc. Many methods use likelihood thresholds to further categorize substitutions as either neutral (tolerant, wild-type, benign) or non-neutral (damaging, pathogenic, deleterious). However, the methods for choosing thresholds vary widely. Some categorization thresholds are established by assessing score distributions across the training sets, and some are determined heuristically.

We further note that thresholding based on experimental training sets can be biased, since different “wet-lab” approaches use different thresholds to classify variant effects and each assay has a technical limit. For example, here we called an experimentally measured two-fold change in repression *neutral*, and everything above that threshold *non-neutral* because biological differences were detected at this level for variants in the quantitative dataset^24^. However, in the semi-quantitative tetramer dataset, everything within ~26-fold of wild-type repression was classified as neutral (“+”)^14^, and variants that repress *better* than wild-type (also non-neutral in our definition) were also considered to be experimentally neutral. Note that the trends of experiment to prediction comparisons were not changed with altered thresholds (Supplementary Fig. 5).

Adding to the overall confusion in the field is the fact that the “effect” definitions are often unclear; *i.e.* manuscripts detailing predictor implementations and/or performance comparisons often mix different effect terms and types of experimental data used for training/development. Thus, at best, most predictors ultimately differentiate between variants of severe effect (*e.g.* those that abolish function or obviously lead to disease) from those that are unlabeled (*e.g.* variants found in healthy populations, 1000 Genomes^25^ or EXAC)^26^ or poorly labeled^27^,^28^ (*e.g.* UniProt^29^ polymorphisms or variants between orthologous sequences).

Here, we showed that thresholding hides the fact that predictors behave differently for rheostat and toggle positions, *i.e.* they generate different score distributions for the two classes. The nearly identical overall performance of all methods – regardless of the effect to be predicted (disease, functional significance, structure, *etc.*) – reveals that current prediction methods are, in essence, trained to differentiate toggle non-neutrals from rheostat neutrals, rather than for the general differentiation of neutral variants from non-neutral ones. This could explain why all predictors appear to have reached an upper threshold in performance.

However, within the rheostat and toggle sets, several methods were able to differentiate neutrals from non-neutrals (Fig. 4b, Supplementary Figs 1b, 2b and 3b). Thus, our results suggest that, if we could reliably label sequence positions as toggle or rheostat, predictions could be improved by using different neutral/non-neutral thresholds for each position class. In particular, this would circumvent the problems that all methods failed to recognize rheostat non-neutrals as having more effect than toggle neutrals and that toggle neutrals scored higher than rheostat neutrals – a difference that is not biologically feasible, as all neutrals are by definition equivalent.

Finally, we note that binary classifications are insufficient to capture variant effects at rheostat positions. Our results show that thresholding prediction scores into binary classes obscures the progressive effects seen for variants at rheostat positions. Nevertheless, the progressive changes are often biologically significant. For the LacI/GalR homologs, progressive functional outcomes translated into progressive and significant changes on bacterial growth rates^24^. Nor can small changes, classified as neutral, be always disregarded: (i) Less than two-fold differences in the function of the tetracycline resistance protein were biologically-adaptive to bacteria in clinically-relevant conditions^30^. (ii) Neutral substitutions in DNA methyl-transferase were deleterious to a host organism under some conditions^31^. (iii) The wide range of normal human phenotypes appears to arise through combinations of weakly non-neutral protein variants^27^. These observations make a compelling argument for building variant-effect predictors that determine a range of outcomes.

For some current algorithms, the magnitude of the prediction scores correlated with the size of the effects. We illustrated this behavior earlier for our method, SNAP^15^,^23^,^27^ and, additionally here, for SNAP2. Further, PROVEAN and PolyPhen-2 showed significant, though weak, correlations for non-neutral variants at rheostat positions. Note that none of the methods was explicitly trained to recognize the severity of effects. Instead, they were trained to differentiate binary effects: neutral from non-neutral. Thus, their prediction scores are indications of a statistical likelihood that a variant of a particular effect will occupy a particular scoring space. Indeed, high impact substitutions at toggle positions, which make up a disproportionately large fraction of the available training sets^7^, were predicted with higher statistical likelihood; *i.e.* toggle non-neutrals score distributions were more dense and significantly higher than rheostat non-neutral scores. In contrast, a statistical likelihood of a variant occupying a neutral scoring space has no equivalent meaning in biology – neutral (no effect) variants cannot be less or more neutral.

We conclude with the acknowledgement that change in protein functionality is not consistently predictive of disease. Regardless of the accuracy of any particular prediction, annotating outcomes is just the first step in a series of inquiries that must be made when trying to map pathogenicity. Each protein must ultimately be considered in the context of its biological role. For example, an interacting protein can change to offset a pathogenic variant to restore normal function^32^. Moreover, functionally deficient proteins may cause disease in some contexts yet protect in others. For example, the variant in hemoglobin that leads to sickle cell anemia in homozygotic humans is protective from malaria for heterozygotes^33^. Thus, variant pathogenicity predictor scores are but one step in modeling the specific mechanisms of disease. Nevertheless, to provide a reliable foundation for quantitative models that predict changes in larger biological systems, we must build consistently-reliable variant-effect predictors.

## Methods

### Experimental characteristics of rheostat positions

In our earlier work^11^, we used a dimeric version of the *E. coli* LacI repressor and synthetic versions of seven other LacI/GalR homologs as hosts for amino acid substitutions at twelve non-conserved positions. All of these positions were located within the linker region that joins the N-terminal DNA binding domain and the allosteric regulatory domain (Fig. 2; PDB 1EFA^34^; positions 46–62). These positions were experimentally shown to be rheostats: At each position, the progressive functional effects of multiple amino acids substitutions were quantified by determining ability to repress transcription of a reporter gene *in vivo*. These studies were extensively validated: For all variants, we confirmed that the protein was expressed at comparable levels, folded, and capable of binding DNA^11^,^35^. We also benchmarked the *in vivo* repression data against *in vitro* biophysical measurements of protein-DNA interactions; in most cases, the repression changes resulted from altered K_d_ for DNA binding^36^,^37^,^38^. Finally, we determined the impact of altered repression on bacterial growth rates to show that the changes were biologically significant^24^.

**Figure 1:**
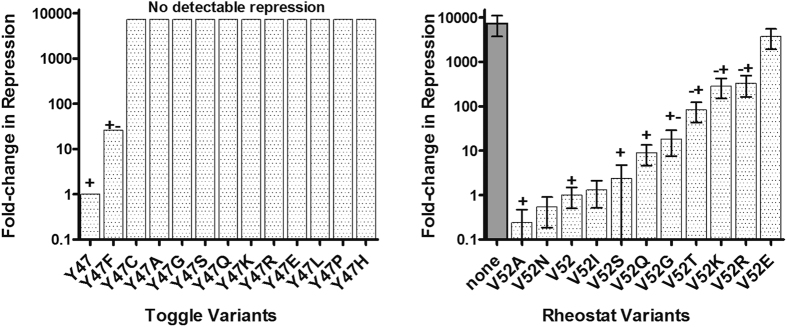
Experimental differentiation of toggle and rheostat positions. The left panel shows an example of a toggle position (tyrosine in position 47): Relative to wild-type (value normalized to 1), most substitutions at LacI position 47 abolish transcription repression of the reporter-gene. The right panel shows an example of a rheostat position (valine in position 52): Variants at this position in LacI exhibit a wide range of repression levels relative to wild-type (value normalized to 1). Data for position 52 (right panel) are adapted from11; the dark gray bar shows the ratio of no-repression (full expression of the reporter gene) to repression by wild-type LacI. Data for position 47 (left panel) were adapted from^14^. Briefly, the earlier study categorized these semi-quantitative data relative to the activity of un-repressed reporter gene (i.e., in the absence of repressor protein). For this figure, we translated the semi-quantitative ranges to the quantitative scale using the “none” value on the right panel.

**Figure 2:**
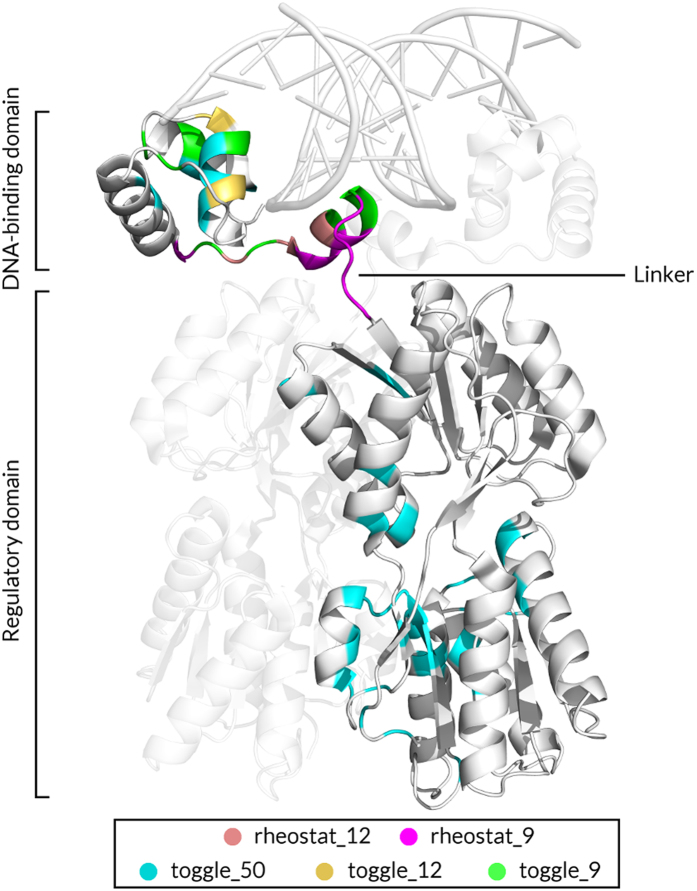
Locations of toggle and rheostat position sets on the structure of the LacI homodimer bound to DNA (PDB 1EFA^34^; visualized with PyMOL)^71^. On one monomer, positions are colored by the sets described in the text. Note that smaller sets are included in the larger sets. For example, *toggle_12* positions are also part of *toggle_50*. Chain B (identical to Chain A) is shown in the background at 50% transparency. DNA is shown as a double helix at the top of the figure.

For the current work, we only used experimental results for *E. coli* LacI (UniProt accession number: P03023). While the synthetic LacI/GalR homologs were critical for disproving the assumptions discussed in the introduction about amino acid interchangeability^11^, these proteins were not naturally evolved and we excluded them to avoid the possibility that they are not properly evaluated by available computational techniques. We also considered whether amino acid substitutions in dimeric LacI had equivalent outcomes in wild-type, tetrameric LacI. The latter is a dimer of dimers, with each dimer serving as a functional unit capable of binding the DNA operator and inducer molecules^39^. Dimeric LacI was created by truncating the C-terminal tetramerization domain^40^. Aside from its lessened ability to simultaneously bind and “loop” two DNA operators^41^, dimeric LacI is extremely similar to tetrameric LacI^40^,^42^. Dimeric LacI was chosen in the 2013 study^11^ so that substitution outcomes could be directly compared with the synthetic homologs, all of which lack a tetramerization domain. For nine of the twelve LacI rheostat positions, the quantitative substitution outcomes experimentally measured for dimeric variants^11^ were in strong agreement with the semi-quantitative *in vivo* measurements previously made for the tetrameric LacI^14^ (Fig. 1, right panel) and with *in vitro* measurements of LacI/DNA variant binding affinities^38^.

Disagreements between the dimeric and tetrameric datasets were only observed for positions 48, 50, and 54. These three positions showed toggle behavior in the tetrameric study, *i.e.* most substitutions abolished function^14^, and rheostat outcomes in the dimeric study^11^. This difference is *opposite* any artifacts expected from truncating the dimerization domain: The tetramerization domain enhances LacI stability relative to the dimer^43^ and tetramer looping enhances repression^44^; either outcome would enhance DNA binding and repression, concealing diminished function of the variants. Thus, we propose the differences between the datasets are due to very low (or zero) LacI protein expression in the tetramer study, which relied upon suppression of amber codons in mutated bacterial strains to create the protein variants^45^. The latter can be an inefficient process that obscures the true outcome of the protein variation. Since the tetramer data are widely used to benchmark computational predictions, developers should be aware that this is a potential experimental bias of this dataset.

For this work, analyses were carried out in parallel using two sets of rheostat positions: all twelve identified in our 2013 study^11^, and the nine that showed agreement between the dimer and tetramer forms of LacI. To determine the categories of neutral/non-neutral, we used the two-fold technical limit of the quantitative repression assay. Thus, variants exhibiting fold-changes in the range of [0.5, 2] relative to wild-type were assigned to the category of *rheostat neutrals*. All other variants were designated as *rheostat non-neutrals*. The fold change used for determining rheostatic behavior was calculated relative to the wild-type repression of 0.124 Miller units. The *rheostat_12* set comprised a total of 103 variants across 12 positions, of which 18 (17%) were neutral and 85 were non-neutral. The *rheostat_9* set comprised 78 variants across 9 positions, of which 18 (23%) were neutral and 60 were non-neutral.

For this study, we considered only the functional impact on repression in the absence of allosteric inducer, since most variants did not show significant changes in allosteric response. For each variant, we re-cast repression in the absence of inducer as fold-change with respect to repression by dimeric wild-type LacI, using equation (1) (Supplementary Table 1):


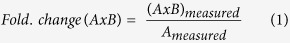


where (*AxB*) stands for a substitution of amino acid *A* by amino acid *B* at position *x* and having the amino acid *A* corresponds to the wild-type protein. Experimental errors associated with the wild-type and variant functional data (standard deviations from the average of ~8 technical and biological replicates) were propagated using equation (2):


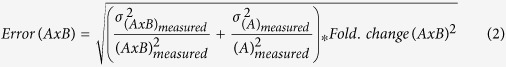


where **σ** is the standard deviation. This equation is derived for correlated variables. For uncorrelated variables, covariance terms present in the original formula equal zero. Solving that formula for the non-squared ratio of two variables results in equation (2).

### Selection and characteristics of toggle positions

Our 2013 study focused on non-conserved positions, and none of the tested LacI positions showed toggle behavior. Thus, to obtain a set of toggle positions for this study, we used the semi-quantitative variant data for tetrameric LacI generated by the Miller lab^14^. In that work, positions 2–329 were substituted with 12–13 amino acids each (Supplementary Table 2) and functional outcomes were broadly grouped into several phenotypes, including those with severe effects (non-neutral) and those with no effects (neutral; Supplementary Table 6). In this work, we defined toggle positions as those with at least 8 severe variants and at most 2 neutral variants. This definition identified 53 toggle positions distributed over the LacI protein. As noted above, rheostat positions 48, 50 and 54 fell in this set of toggles positions. As noted above, we hypothesized that these results are due to extremely low protein concentrations, perhaps related to inefficient suppression of the amber codon, and we thus excluded these positions to yield the final *toggle_50* set (618 variants, 26 neutral and 592 non-neutral).

To create a toggle set of comparable size to the rheostat set, we selected a subset of toggle positions based on the availability of neutral variants and structural proximity to rheostat positions: First, we selected all positions within the DNA-binding domain with at least one neutral variant (10, 13, 30, 49). Second, toggle positions 47, 49, 53, 56, and 57 were chosen because they interdigitate with rheostat positions in the linker region (46–62). Of the remaining 13 toggles within the DNA-binding domain, we selected positions 16, 18, 21, and 22. The resulting *toggle_12* set comprised 145 variants across 12 positions, of which 4 (2.8%) were neutral. We also designated the *toggle_9* set (*toggle_12* minus variants at positions 16, 21 and 22), which comprised 109 variants (4 *toggle neutrals* and 105 *non-neutrals*) across 9 positions.

In Results, we labeled the *rheostat_9 vs. toggle_9* comparison as the *stringent* set (see Fig. 4, Supplementary Fig. 1). For a more comprehensive analysis, we also computed results for *complete* (Supplementary Fig. 2) and *extended* (Supplementary Fig. 3) sets; which included the *rheostat_12* set and, respectively, the *toggle_12* or the full *toggle*_*50* set (Supplementary Table 2). By using the three comparison sets, we hoped to minimize the impact of mis-assigned toggle positions due to poor protein expression.

### Variant-effect prediction algorithms

**Figure 3:**
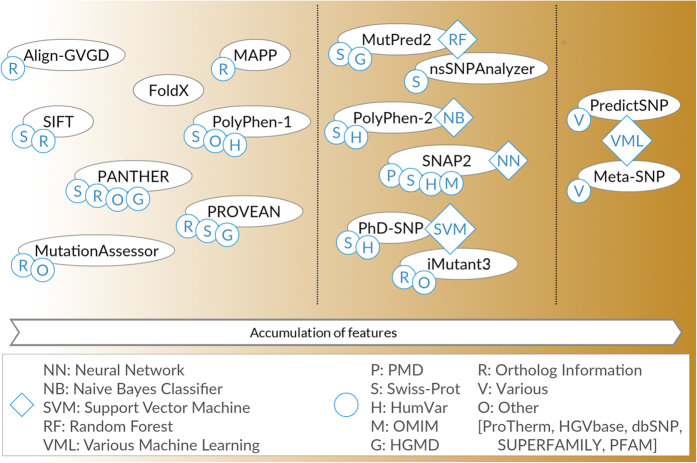
Variant-effect predictors vary in features and development data used. The 16 publicly available variant-effect prediction algorithms can be broadly grouped by use of (i) basic biological principles and evolutionary information, (ii) pattern recognition techniques and machine learning, and (iii) meta/ensemble predictors.

**Figure 4:**
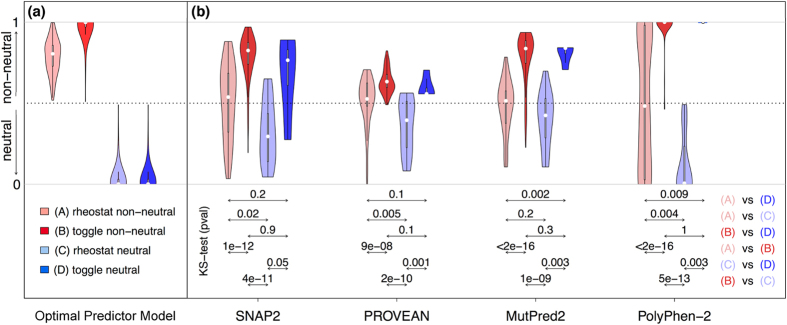
Distributions of variant scores from continuous prediction methods differ between rheostat and toggle positions (*stringent* set). Panel (a) shows the distributions expected from an ideal variant-effect predictor, while panel (b) shows the distributions determined for neutral and non-neutral variants at both rheostat and toggle positions in the stringent set. These four predictors were selected on the basis of top performance in differentiating rheostat non-neutrals from rheostat neutrals. Results for all other predictors are in Supplementary Fig. 1. The violin plot is an augmented box plot where the width at any given Y-axis value indicates the probability density of the data (median, white circles; interquartile range, box outline). The p-values in the legend are from a Kolmogorov-Smirnov (KS) test, indicating whether a method can significantly distinguish between the two distributions pointed to by the respective arrows. Results from the complete and extended sets are in Supplementary Figs 2 and 3.

**Figure 5:**
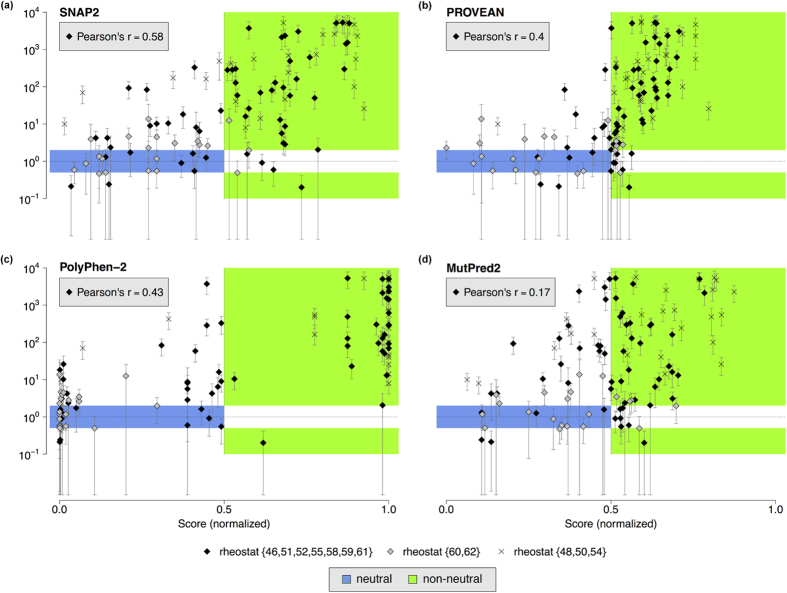
Correlation between experimentally measured fold-changes and predicted variant-effect scores. Panels (a) SNAP2; (b) PROVEAN; (c) MutPred2; (d) PolyPhen-2 show the relationship of the computationally and experimentally derived scores. For each variant at all rheostat positions, fold-change in repression relative to wild-type LacI is shown on log scale (Y axis), whereas predicted scores are normalized to the linear range [0, 1] (X axis). The blue area depicts the scores expected for neutral variants (fold-change between 0.5 and 2.0); the green area depicts scores expected for non-neutral variants. The Pearson product-moment correlation coefficient (Pearson’s r) is given for the *rheostat_9* set. Results from other predictors are in Supplementary Fig. 4.

To predict variant effects at rheostat and toggle positions, we used 16 publicly available computational methods (Fig. 3, Supplementary Table 7). These were selected to cover a wide variety of computational techniques and training sets. Note that not all publications explicitly mention what is meant by the word “effect.” Some predict disease variants, others focus on evolutionary conservation or evolutionary fitness, and still others evaluate functional or structural impacts. To be able to use all tools, here we broadly use the term *outcome* without further identifying differences between methods. For all methods that require a 3D structure, we used 1EFA (PDB ID: 1EFA)^34^, consisting of dimeric LacI bound to DNA operator and an “anti-inducer” allosteric ligand. All reported graphs/statistics were generated using R^46^.

1. SIFT^22^ uses PSI-BLAST^47^ (position-specific iterated Basic Local Alignment Search Tool; BLAST) to query a sequence database (*e.g.* NCBInr)^48^ and generates a position-specific scoring matrix (PSSM) based on the retrieved sequences. Note that we used SIFT with a manually curated MSA (Supplementary File 1)^49^ rendering the initial PSI-BLAST query obsolete. Combined with known generic likelihoods of amino acid substitutions (BLOSUM62 substitution matrix), this approach allows estimating probabilities of effect for any position-specific amino acid substitution.
2. PROVEAN^16^ uses BLAST^50^ to collect homologous and distantly related sequences from NCBInr, which are then clustered by sequence identity. To measure the effect of a variation, the algorithm calculates the average divergence score between the cluster sequences and the query sequence using the BLOSUM62 substitution matrix.
3. PANTHER^19^ uses BLAST to identify the best match to the input query in the PANTHER database^51^. This matched protein is linked to a pre-computed phylogenetic tree of the specific protein family. To evaluate the effect of the substitution, the mutated residue is traced back through increasingly older ancestral proteins in this tree.
4. MutationAssessor^52^ uses BLAST to query the UniProt and identify related protein sub-families, which are used to extract characteristic conservation patterns. The latter are used to calculate the effect of mutating a specific residue in a protein family and, separately, in each of its sub-families.
5. FoldX (PositionScan)^53^ uses protein 3D structures and an empirical force field to evaluate the effects (free energy changes) due to variation.
6. Align-GVGD^54^ is an extension of the Grantham Difference^55^, combining a conservation score based on a given MSA (Grantham Variation) with a measure of the biochemical difference between the mutant and the wild-type amino acids (Grantham Deviation).
7. MAPP^56^ constructs a phylogenetic tree based on substitution frequency per site within an MSA of orthologs or closely related paralogs. Topology and branch lengths of the tree are used to calculate weights for each sequence respectively. These weights are used to generate an alignment summary that is interpreted using a matrix of physiochemical properties resulting in an estimate of the physiochemical constraints on each position of the MSA. Deviations from these constraints are calculated for each position of the query sequence and transformed into an effect prediction score.
8. PolyPhen-1^57^ classifies variants via empirically derived rules using various sequence-based characteristics of the substitution site (*e.g.* UniProt annotations), along with structure and homology descriptors.
9. PolyPhen-2^17^ trains a Naïve Bayes classifier on HumDiv (set of single amino acid substitutions from UniProt known to cause human Mendelian diseases and non-damaging variants found in closely related mammalian homologs) and HumVar^58^ (set of disease- and neutral polymorphism-annotated single amino acid substitutions of human proteins from Swiss-Prot)^29^ variants. PolyPhen-2 uses structure-based (*e.g.* accessible surface area and conformational mobility of the wild-type amino acid residue) and sequence-based features (*e.g.* MSA-based conservation and depth, CpG context and residue volume).
10. SNAP2^15^ uses an artificial neural network, trained on experimentally-obtained variant functional effect data, with a variety of precomputed biochemical and evolutionary amino acid substitution rules, as well as conservation and predicted sequence-derived protein features, *e.g.* secondary structure and solvent accessibility.
11. PhD-SNP^58^ applies a decision tree to predict effects either using a profile based support vector machine (SVM; sequence profile calculated by BLAST against UniRef90) or by a single sequence based SVM. Training data are extracted from Swiss-Prot (disease *vs.* polymorphism variants) and enriched with OMIM^59^ annotations.
12. iMutant3^58^,^60^ offers different SVMs to predict (i) stability changes, using SVMs trained on the ProTherm database^61^, from sequence only or from structural information, and (ii) disease associated variants from sequence only using the PhD-SNP prediction pipeline.
13. nsSNPAnalyzer^62^ uses a random forest classifier, trained on a curated dataset of variants (ModSNP)^63^ using (i) variant structural environment, (ii) position conservation within the MSA, and (iii) similarity between variant and original amino acid. If no structural information is provided, the ASTRAL database^64^ is queried for a homolog structure.
14. PredictSNP^65^ is a meta-predictor incorporating eight methods (MAPP, nsSNPAnalyzer, PANTHER, PhD-SNP, PolyPhen-1/-2, SIFT, SNAP2) into a consensus classifier based on a majority vote weighted by the method-specific confidence scores. PredictSNP is trained on a benchmark dataset compiled from five different sources (training datasets of four variant-effect prediction tools not selected for the PredictSNP pipeline: SNPs&GO, MutPred^18^, PON-P and HumVar; the fifth source is a subset of UniProt variants). Testing datasets are derived from the Protein Mutant Database^66^ (PMD) and from experimental studies.
15. Meta-SNP^67^ is a random forest-based binary classifier meta-predictor, combining the predictions of four methods (SNAP2, SIFT, PANTHER, PhD-SNP) and four features extracted from the PhD-SNP protein sequence profile; the training dataset is derived from Swiss-Prot (disease *vs.* polymorphism variants).
16. MutPred2 (unpublished; the successor of MutPred)^18^ consists of an ensemble of bagged neural networks, trained on amino acid substitutions from HGMD^68^, Swiss-Prot, dbSNP^69^, and ortholog alignments. In addition to sequence, conservation, and physicochemical features in and around the variant position, MutPred2 uses predictions of change due to amino acid variation in over 50 local structural and functional properties (*e.g.* post-translational modification sites, macromolecular binding, among others).

For all variants in the LacI rheostat and toggle sets, predictions were generated using the 16 algorithms listed above. When no publicly available web-service was present, prediction methods were installed and run locally. Input parameters were set to default values. To obtain comparable predictions between the different algorithms, all predictor scores were transformed and normalized: Some tools provide a probability or some other score for the likelihood of variant non-neutrality. For these, we converted pre-defined, method-specific binary thresholds to a value of 0.5 and normalized the neutral and non-neutral score ranges separately to [0, 0.5] and (0.5, 1], respectively. For methods that predict the classes of functional outcomes, scores were assigned manually. Details of scoring and thresholds used for normalization are as follows:

1. SNAP2 scores are [−100, 100], threshold at 0, neutrals below threshold.
2. SIFT scores are [0, 1], threshold at 0.05, neutrals above threshold. Scores were reversed (equation (3) prior to normalization.
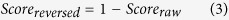
3. MutationAssessor score range was not defined by the authors, but available data^52^ suggests a [−4, 5] range, which we use in normalization; default threshold is 1.9, neutrals below threshold. Note, however, in this work we used a threshold of 0.8, as described in ref. 16, to more accurately differentiate neutrals.
4. PROVEAN score range was not defined by the authors, but predicted scores for our variants occurred in the [−14.875, 1.908] range, which we use in normalization; threshold at −2.5, neutrals above threshold.
5. MAPP scores are [0, 1], threshold at 0.5, neutrals above threshold. Scores were reversed using equation (3).
6. iMutant3 score range was not defined by the authors, but predicted scores for our variants occurred in the [−3.5, 0.63] range, which we use in normalization; threshold at −0.5, neutrals above threshold. Note that we used this threshold to transform predictions into a binary form.
7. FoldX (PositionScan) score range is not defined by the authors, but predictions for free energy changes below 0.05 kcal/mol (neutrals) are not reported. The maximum predicted score for our variants was 3.76102. Here we assigned a score of 0 to missing predictions, resulting in [0, 3.76102] range, and set the threshold at 0.5 (as in iMutant, above), neutrals below the threshold. Note that we used this threshold (as in iMutant, above), to transform predictions into a binary form.
8. PolyPhen-1 classifications of [benign, possibly damaging, probably damaging], were converted to [0, 0.5, 1]
9. Align-GVGD classifications are [C0, C15, C25, C35, C45, C55, C65], assigned to corresponding risk estimates, ranging [1.16, 3.12]^70^. The authors did not define the threshold, but C0 was suggested to be the only neutral class. Thus, threshold was set at the corresponding risk estimate of 1.16, neutrals below threshold. Note that we used this threshold to transform predictions into a binary form.
10. PolyPhen-2 scores are [0, 1], threshold at 0.92, neutrals below threshold.

 11-14. MutPred2, PANTHER, PhD-SNP, and Meta-SNP obtain probability scores, which range [0, 1] with a threshold at 0.5, neutrals below threshold. Note that scores for PANTHER and PhD-SNP reported here were each obtained from meta-predictors (PredictSNP and Meta-SNP, respectively).

 15,16. nsSNPAnalyzer and PredictSNP are binary classifiers [neutral, non-neutral], which were converted to [0, 1].

To analyze the performance of prediction tools in differentiating rheostat and toggle position non-neutrals and neutrals, we applied the Kolmogorov-Smirnov (KS) test (two-sided) for continuous predictors and Fisher exact T-test (two-sided) for binary predictors. To compare the correlation between experimentally-measured fold-changes for rheostat variants and predicted variant-effect scores, we computed the Pearson product-moment correlation coefficient (Pearson’s r). For this analysis, we made two assumptions: (i) In addition to the variants with diminished repression, five variants showed enhanced repression relative to wild-type (fold-change values less than 0.5) and thus were treated as non-neutral. We used the reciprocal of the fold-change to correlate them to prediction scores. (ii) Neutral variants are, by definition, all equivalent to wild-type. However, the continuous predictors usually assign a range of scores that fall below their neutrality threshold (here normalized to 0.5). Thus, we assigned all neutrally predicted variants a score of 0.5.

## Additional Information

**How to cite this article:** Miller, M. *et al*. Computational predictors fail to identify amino acid substitution effects at rheostat positions. *Sci. Rep.* **7**, 41329; doi: 10.1038/srep41329 (2017).

**Publisher's note:** Springer Nature remains neutral with regard to jurisdictional claims in published maps and institutional affiliations.

## Supplementary Material

Supplementary File 1

Supplementary Information

Supplementary Table 1

Supplementary Table 2

Supplementary Table 3

Supplementary Table 4

Supplementary Table 7
