# Supplementary material for: Computational predictors fail to identify amino acid substitution effects at rheostat positions
Source: Sci Rep. 2017 Jan 30;7:41329. doi: 10.1038/srep41329 (PMC5278360; doi:10.1038/srep41329)
Supplement: Supplementary Information [file srep41329-s3.pdf]

Supporting online material for:

Computational predictors fail to identify amino acid substitution effects at rheostat positions

Miller M, Bromberg Y and Swint-Kruse L

Table of Contents for Supporting Online Material

Table 1 – Experimentally-measured, quantitative variant outcomes generated for rheostat positions in dimeric LacI by the Swint-Kruse laboratory.

Table 2 – Experimentally-measured, semi-quantitative outcomes for variants of tetrameric LacI generated by the Miller laboratory.

Table 3 – Predictions for variants at rheostat positions.

Table 4 – Predictions for variants at toggle positions.

Table 5 – Correlation of repressor functional change and predicted variant-effect scores compared for *rheostat_9* and *rheostat_12* sets.

Table 6 – Grouping of tetramer LacI phenotypes into functional outcomes used for this study.

Table 7 – Comprehensive overview of the characteristics for variant-effect predictors.

File 1 – Multiple sequence alignment of the LacI/GalR family.

Figure 1 – Distributions of variant scores from continuous and binary prediction methods for the *stringent* set are different between rheostats and toggles.

Figure 2 – Distributions of variant scores from continuous and binary prediction methods for the *complete* set are different between rheostats and toggles.

Figure 3 – Distributions of variant scores from continuous and binary prediction methods for the *extended* set are different between rheostats and toggles.

Figure 4 – Correlation between experimentally measured fold-changes and predicted variant-effect scores for rheostat variants.

Figure 5 – Trends in distributions of variant scores do not change with altered neutrality threshold.

Short description for additional Files in the Supporting Online Material

Table 1 – Experimentally-measured, quantitative variant outcomes generated for rheostat positions in dimeric LacI by the Swint-Kruse laboratory. Activity of the β-galactosidase reporter gene ("Liquid culture Bgal") was measured for *E. coli* that expressed variants of the lactose repressor protein (LacI)[1]. Amino acid substitutions were created at 12 non-conserved positions in dimeric LacI ("lac-11)" by site-directed mutagenesis at the codon of interest in a plasmid carrying the *lacI* gene. Reporter activity was determined in the absence ("-") and presence of allosteric inducer ("+inducer"). The top row contains the value for wild-type dimeric LacI, which was used to calculate fold-change for all variants. Note that low activity and fold change values correspond to strong transcription repression, whereas high activity and fold change values correspond to weak repression.

Table 2 – Experimentally-measured, semi-quantitative outcomes for variants of tetrameric LacI generated by the Miller laboratory. Activity of the β-galactosidase reporter gene was measured in *E. coli* strains co-expressing variants of tetrameric

LacI[2]. Mutations were generated by (i) changing the codon of interest in the *lacI* gene to the amber codon and (ii) using 12-13 *E. coli* amber-suppression strains to substitute the desired amino acid. These data were used in the current study to identify toggle positions in LacI, as indicated in the right-hand columns and as described in Methods of the main text. Abbreviations for repression phenotypes are as follows: +, repression > 200 fold relative to activity of the unrepressed reporter gene; + −, repression ranges [20,200] fold; − +, repression ranges [4,20] fold; −, repression < 4. Abbreviations for induction phenotypes are as follows: s, no induction; ws, weak induction; vws, very weak induction. Additional phenotypes: c, cold sensitive mutants; h, heat sensitive mutants; r, mutants with reverse induction profile.

Table 3 – Variant-effect predictions for all LacI rheostat variants, raw and normalized scores. The first two columns of numbers contain the experimentally-determined fold change (with propagated error) for each of the variants.

Table 4 – Variant-effect predictions for all LacI toggle variants, raw and normalized scores.

Table 7 – Comprehensive overview of the characteristics for variant-effect predictors. Specific versions used to generate Supplementary Tables S5 and S6 are given, in addition to common characteristics of the various algorithms.

File 1 – Multiple Sequence Alignment of LacI and 350 related sequences, manually curated by the Swint-Kruse lab[3].

Material

Prediction Method	Pearson's r *rheostat_9*	Pearson's r *rheostat_12*
SNAP2	0.58	0.61
PROVEAN	0.4	0.41
PolyPhen-2	0.43	0.45
MutPred2	0.17	0.32

Table 5. Correlation of repressor functional change and predicted variant-effect scores compared for *rheostat_9* and *rheostat_12* sets. The four methods exhibiting statistically-significant differentiation of rheostat neutrals from non-neutrals show the same trends for correlation of measured fold-changes and prediction scores within both *rheostat_9* and *rheostat_12* sets.

Effect on function	Phenotypes in tetrameric LacI
Neutral	+ , +c, +h
Intermediate	+ ws, + vws, + -, + - h, + - ws, + - vws, - +, - + ws, - + vws
Severe	-, - + s, +s, + - s

Table 6. Grouping of tetramer LacI phenotypes into functional outcomes used for this study. Abbreviations for repression phenotypes are as follows: +, repression > 200 fold relative to activity of the unrepressed reporter gene; + −, repression ranges [20,200] fold; − +, repression ranges [4,20] fold; −, repression < 4. Abbreviations for induction phenotypes are as follows: s, no induction; ws, weak induction; vws, very weak induction. Additional phenotypes: c, cold sensitive mutants; h, heat sensitive mutants; r, mutants with reverse induction profile.

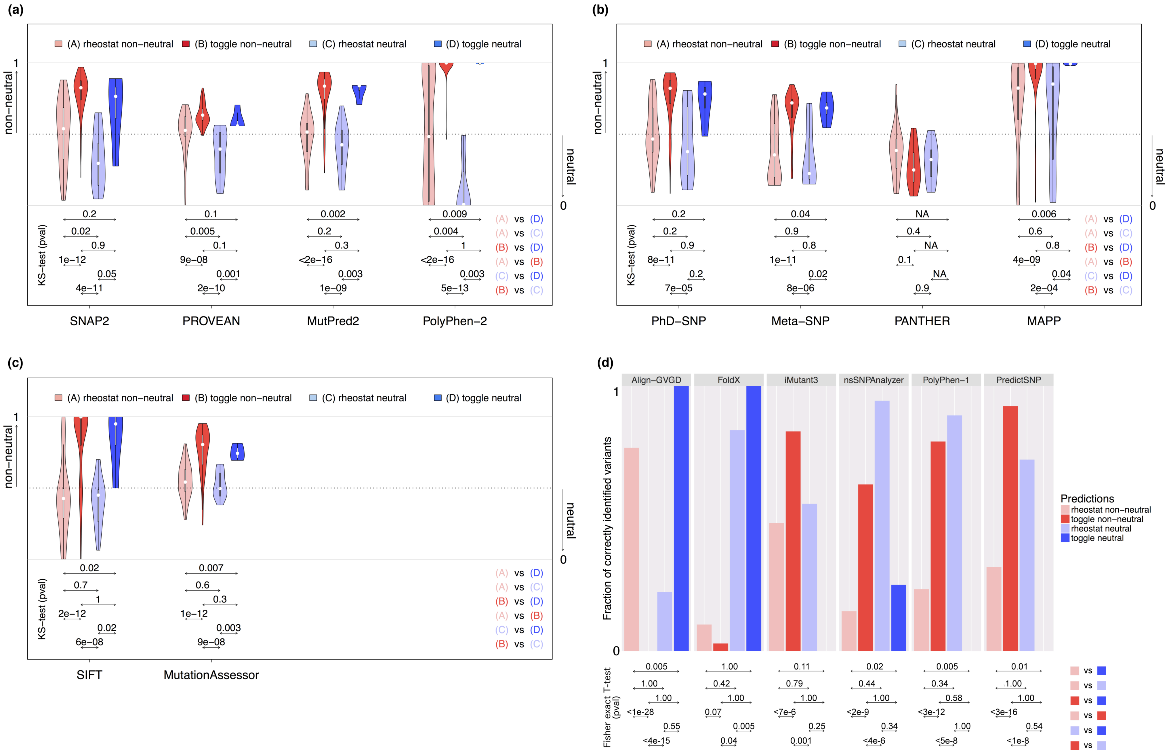

Figure 1. Distributions of variant scores from continuous and binary prediction methods for the *stringent* set differ between rheostat and toggle positions. Panels (a) to (c) show the distributions for continuous predictors, determined for neutral and non-neutral variants at both rheostat and toggle positions. The violin plot is an augmented box plot where the width at any given Y-axis value indicates the probability density of the data (median, *white circle*s; interquartile range, *box outline*). The p-values below the plots are from a Kolmogorov-Smirnov (KS) test, indicating whether a method can significantly distinguish between the two distributions pointed to by the respective arrows. Panel (d) shows the fraction of correctly-predicted rheostat and toggle non-neutral and neutral variants for binary predictors. The p-values below the plot are from a Fisher exact T-test, indicating whether a method can significantly distinguish between the two distributions pointed to by the respective arrows.

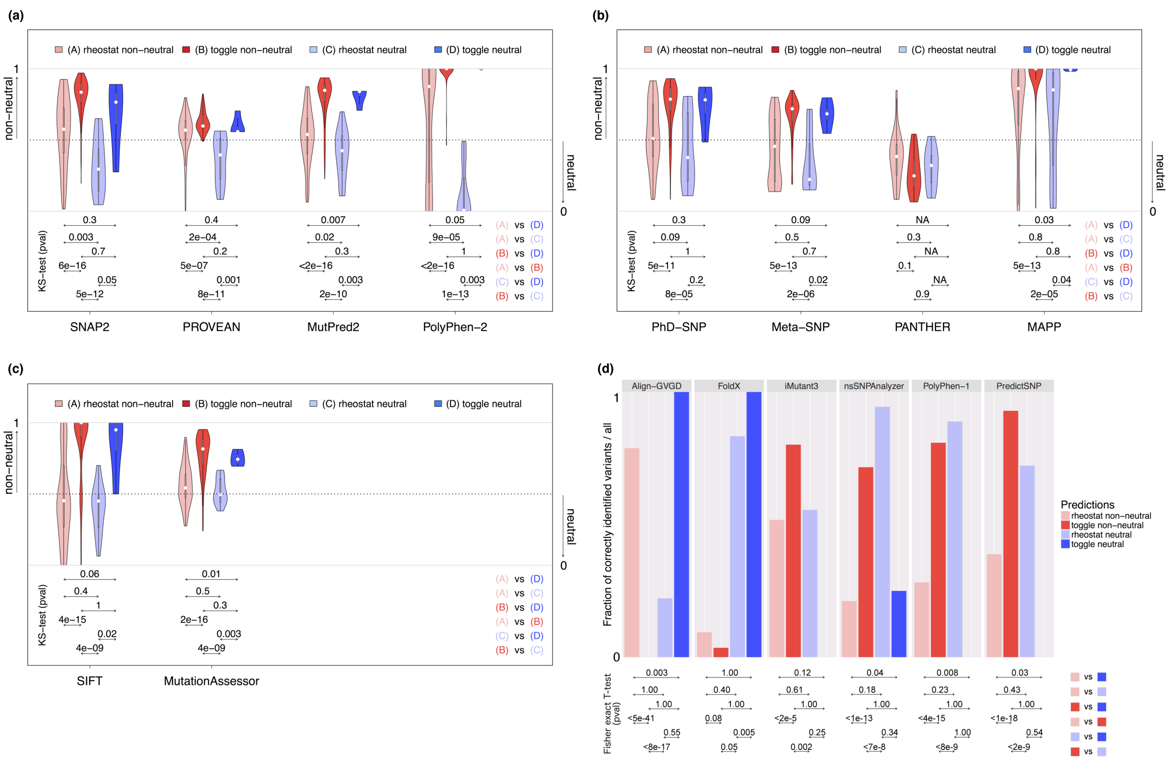

Figure 2. Distributions of variant scores from continuous and binary prediction methods for the *complete* set differ between rheostat and toggle positions. Panels (a) to (c) show the distributions for continuous predictors, determined for neutral and non-neutral variants at both rheostat and toggle positions. The p-values below the plots are from a Kolmogorov-Smirnov (KS) test, indicating whether a method can significantly distinguish between the two distributions pointed to by the respective arrows. Panel (d) shows the fraction of correctly-predicted rheostat and toggle non-neutral and neutral variants for binary predictors. The p-values below the plot are from a Fisher exact T-test, indicating whether a method can significantly distinguish between the two distributions pointed to by the respective arrows.

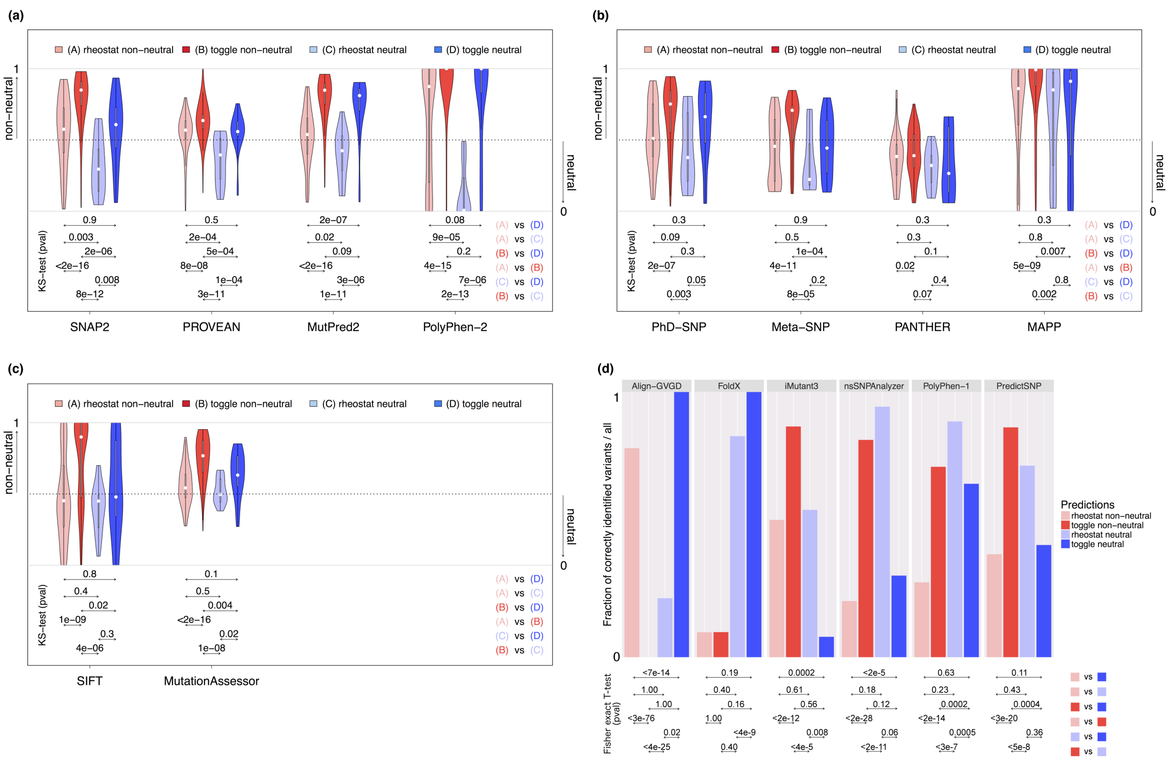

Figure 3. Distributions of variant scores from continuous and binary prediction methods for the *extended* set differ between rheostat and toggle positions. Panels (a) to (c) show the distributions for continuous predictors, determined for neutral and non-neutral variants at both rheostat and toggle positions. The p-values below the plots are from a Kolmogorov-Smirnov (KS) test, indicating whether a method can significantly distinguish between the two distributions pointed to by the respective arrows. Panel (d) shows the fraction of correctly-predicted rheostat and toggle non-neutral and neutral variants for binary predictors. The p-values below the plots are from a Fisher exact T-test, indicating whether a method can significantly distinguish between the two distributions pointed to by the respective arrows.

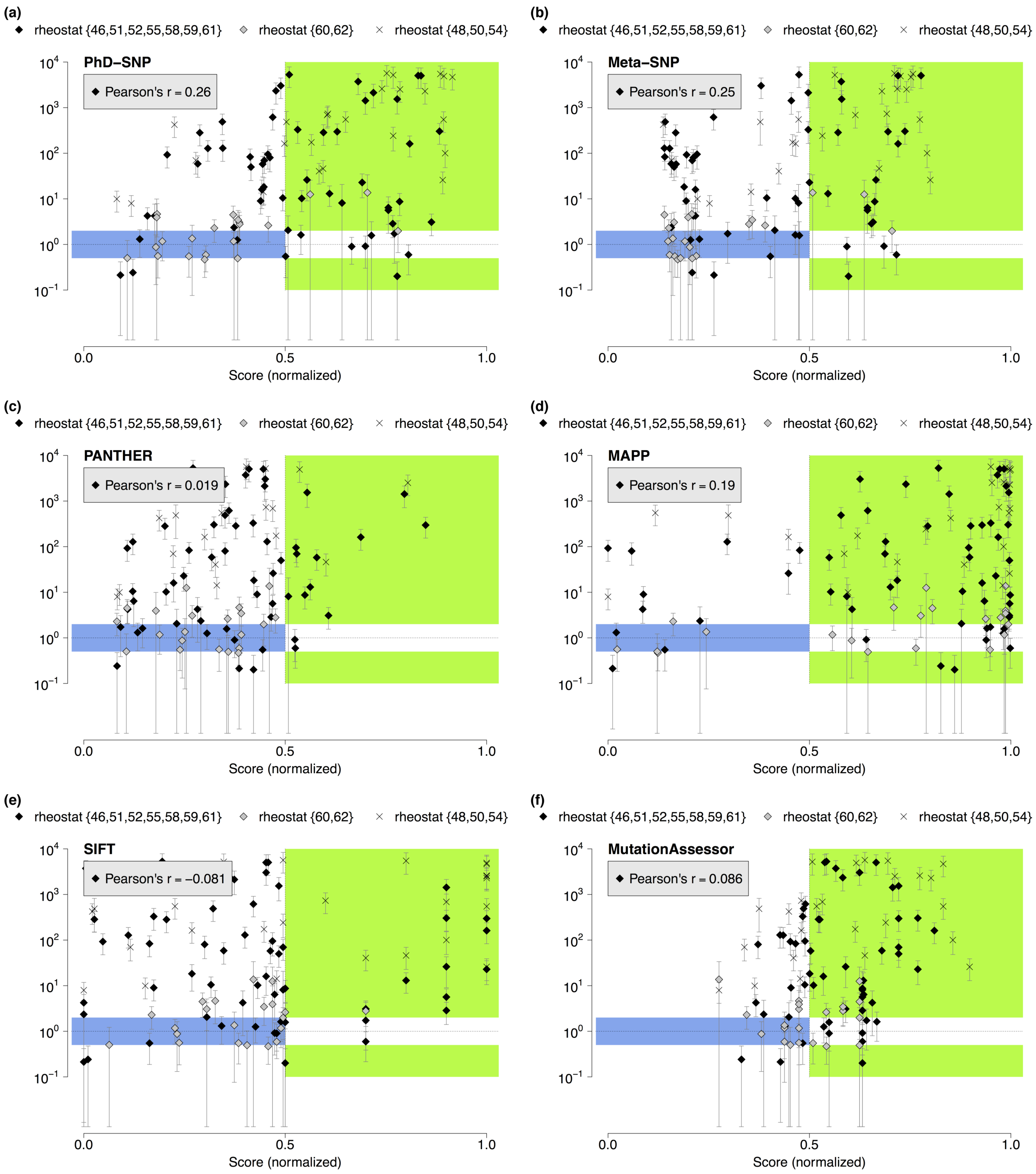

Figure 4. Correlation between experimentally measured fold-changes and predicted variant-effect scores for rheostat variants. Panels (a) to (f) show the relationship of the computationally and experimentally derived scores. For each variant at all rheostat positions, fold-change in repression relative to wild-type LacI is shown on log scale (Y axis), whereas predicted scores are normalized to the linear range [0,1] (X axis). The blue area depicts the scores expected for neutral variants (fold-change between 0.5 and 2.0); the green area depicts scores expected for non-neutral variants. The Pearson product-moment correlation coefficient (Pearson's r) is given for the *rheostat_9* set.

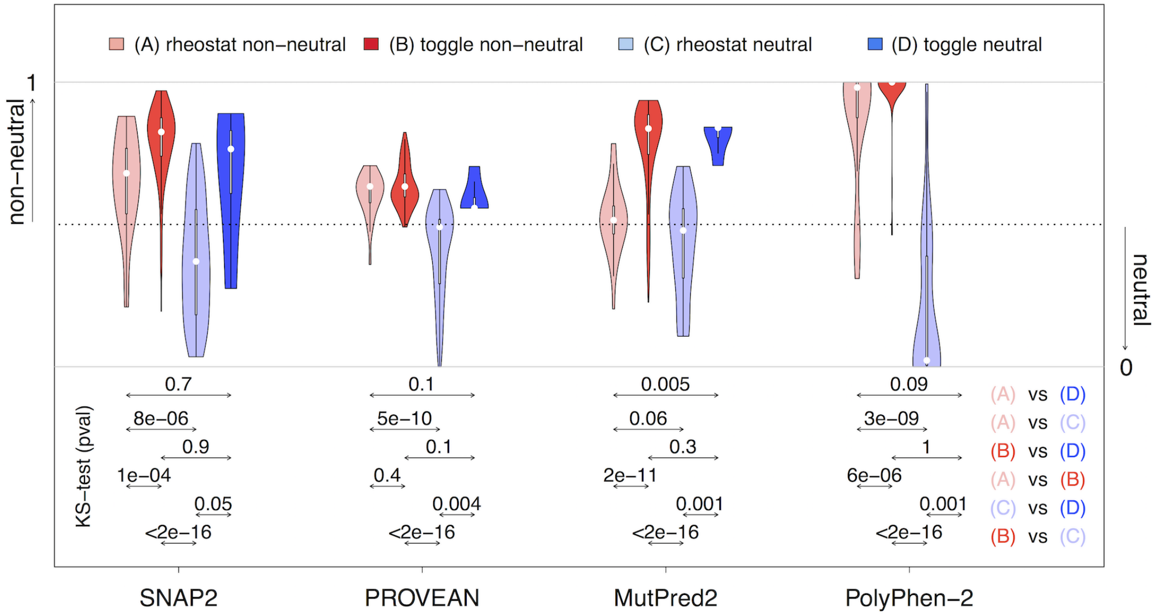

Figure 5. Trends in distributions of variant scores do not change with altered neutrality threshold. Shown is the distributions for continuous predictors using an extended neutrality threshold, determined for neutral and non-neutral variants at both rheostat and toggle positions for the *stringent* set. The p-values below the plots are from a Kolmogorov-Smirnov (KS) test, indicating whether a method can significantly distinguish between the two distributions pointed to by the respective arrows. The extended neutrality threshold derives from altering the default threshold (fold-change between 0.5 and 2.0) by factor 20 (fold-change between 0.05 and 40.0). The trends of experiment to prediction comparisons do not change with regards to the default threshold.

References for Supporting Online Material

Table 1: [1]

Table 2: [2]

Table 3: [4, 5, 6, 7, 8, 9, 10, 11, 12, 13, 14, 15, 16, 17, 18]

Table 4: [4, 5, 6, 7, 8, 9, 10, 11, 12, 13, 14, 15, 16, 17, 18]

Table 5,6,7: none

File1: Manually-curated Multiple Sequence Alignment[3]

1. Meinhardt S, Manley MW, Jr., Parente DJ, Swint-Kruse L. Rheostats and toggle switches for modulating protein function. *PLoS One* **8**, e83502 (2013).

2. Suckow J, Markiewicz P, Kleina LG, Miller J, Kisters-Woike B, Muller-Hill B. Genetic studies of the Lac repressor. XV: 4000 single amino acid substitutions and analysis of the resulting phenotypes on the basis of the protein structure. *J Mol Biol* **261**, 509-523 (1996).

3. Tungtur S, Parente DJ, Swint-Kruse L. Functionally important positions can comprise the majority of a protein's architecture. *Proteins* **79**, 1589-1608 (2011).

4. Hecht M, Bromberg Y, Rost B. Better prediction of functional effects for sequence variants. *BMC Genomics* **16 Suppl 8**, S1 (2015).

5. Ng PC, Henikoff S. Predicting deleterious amino acid substitutions. *Genome Res* **11**, 863-874 (2001).

6. Choi Y, Sims GE, Murphy S, Miller JR, Chan AP. Predicting the functional effect of amino acid substitutions and indels. *PLoS One* **7**, e46688 (2012).

7. Tang H, Thomas PD. PANTHER-PSEP: predicting disease-causing genetic variants using position-specific evolutionary preservation. *Bioinformatics*, (2016).

8. Reva B, Antipin Y, Sander C. Predicting the functional impact of protein mutations: application to cancer genomics. *Nucleic Acids Res* **39**, e118 (2011).

9. Schymkowitz J, Borg J, Stricher F, Nys R, Rousseau F, Serrano L. The FoldX web server: an online force field. *Nucleic Acids Res* **33**, W382-388 (2005).

10. Mathe E, Olivier M, Kato S, Ishioka C, Hainaut P, Tavtigian SV. Computational approaches for predicting the biological effect of p53 missense mutations: a comparison of three sequence analysis based methods. *Nucleic Acids Res* **34**, 1317-1325 (2006).

11. Stone EA, Sidow A. Physicochemical constraint violation by missense substitutions mediates impairment of protein function and disease severity. *Genome Res* **15**, 978-986 (2005).

12. Ramensky V, Bork P, Sunyaev S. Human non-synonymous SNPs: server and survey. *Nucleic Acids Res* **30**, 3894-3900 (2002).

13. Capriotti E, Fariselli P, Calabrese R, Casadio R. Predicting protein stability changes from sequences using support vector machines. *Bioinformatics* **21 Suppl 2**, ii54-58 (2005).

14. Adzhubei IA*, et al.* A method and server for predicting damaging missense mutations. *Nat Methods* **7**, 248-249 (2010).

15. Bao L, Zhou M, Cui Y. nsSNPAnalyzer: identifying disease-associated nonsynonymous single nucleotide polymorphisms. *Nucleic Acids Res* **33**, W480-482 (2005).

16. Bendl J*, et al.* PredictSNP: robust and accurate consensus classifier for prediction of disease-related mutations. *PLoS Comput Biol* **10**, e1003440 (2014).

17. Capriotti E, Altman RB, Bromberg Y. Collective judgment predicts disease-associated single nucleotide variants. *BMC Genomics* **14 Suppl 3**, S2 (2013).

18. Capriotti E, Calabrese R, Casadio R. Predicting the insurgence of human genetic diseases associated to single point protein mutations with support vector machines and evolutionary information. *Bioinformatics* **22**, 2729-2734 (2006).
